# Deep Learning-Based Myoelectric Potential Estimation Method for Wheelchair Operation

**DOI:** 10.3390/s22041615

**Published:** 2022-02-18

**Authors:** Shimpei Aihara, Ryusei Shibata, Ryosuke Mizukami, Takara Sakai, Akira Shionoya

**Affiliations:** 1 Department of Sport Science, Japan Institute of Sports Sciences, 3-15-1 Nishigaoka, Kita-ku, Tokyo 115-0056, Japan; 2 School of Creative Science and Engineering, Waseda University, Wasedamachi-27, Shinjuku-ku, Tokyo 169-8050, Japan; 3 Graduate School of Information and Management Systems Engineering, Nagaoka University of Technology, 1603-1, Kamitomioka, Nagaoka, Niigata 940-2188, Japan; s193373@stn.nagaokaut.ac.jp (R.M.); s181038@stn.nagaokaut.ac.jp (T.S.); shionoya@vos.nagaokaut.ac.jp (A.S.)

**Keywords:** myoelectric potential, deep learning, camera, inertia sensor

## Abstract

Wheelchair sports are recognized as an international sport, and research and support are being promoted to increase the competitiveness of wheelchair sports. For example, an electromyogram can observe muscle activity. However, it is generally used under controlled conditions due to the complexity of preparing the measurement equipment and the movement restrictions imposed by cables and measurement equipment. It is difficult to perform measurements in actual competition environments. Therefore, in this study, we developed a method to estimate myoelectric potential that can be used in competitive environments and does not limit physical movement. We developed a deep learning model that outputs surface myoelectric potentials by inputting camera images of wheelchair movements and the measured values of inertial sensors installed on wheelchairs. For seven subjects, we estimated the myoelectric potential during chair work, which is important in wheelchair sports. As a result of creating an in-subject model and comparing the estimated myoelectric potential with the myoelectric potential measured by an electromyogram, we confirmed a correlation (correlation coefficient 0.5 or greater at a significance level of 0.1%). Since this method can estimate the myoelectric potential without limiting the movement of the body, it is considered that it can be applied to the performance evaluation of wheelchair sports.

## 1. Introduction

Sports promote mental and physical development, enrich humanity, and play an important role in living a healthy life. For persons with disabilities, sports can be an important component of medical rehabilitation. In addition, sports can provide lifelong recreation and can be played at various levels, e.g., the Paralympics. In Japan, especially in competitive sports, interest in sports for the disabled increased with the opening of the Olympic and Paralympic Tokyo 2020 Games. In particular, wheelchair sports account for 50% of the competitions held at the Paralympic Games, e.g., tennis, basketball, athletics marathons, short/medium/long-distance running and relays, badminton, rugby, and table tennis. It is recognized as an international sport. In recent years, the rules for wheelchair sports, which have been disparate in each country, have been established as international rules, and global competitiveness has advanced significantly. In wheelchair sports, a person and their wheelchair play together; thus, the chair work that controls the wheelchair similar to part of the body greatly affects the competitiveness of wheelchair athletes. The relationship between chair work and performance has been reported previously with a focus on wheelchair basketball, wheelchair tennis, and wheelchair rugby [1,2,3].

There is a kinetic approach that focuses on the changes and shapes of body parts [4]. In this approach, motion capture is a typical tool. Optical motion capture is widely considered the gold standard for motion capture because it is the most accurate method to track the kinematics of human movement [5]. For example, Franchin et al. reported that it was possible to identify the technology by measuring the trajectory of the wrist and elbow during a push motion using optical motion capture for wheelchair rugby [6]. However, optical motion capture can only be used in a narrow observation area, and the calibration procedure requires time and skill; thus, it is primarily used in laboratory settings. Therefore, using computer vision to analyze video images recorded at competition practice sites is increasing because there are few restrictions on the measurement location, the measurement process is easy, and the cost is low [7,8]. For the same reason, the use of wearable inertial sensors is also increasing in the field of competition [9,10].

There is also a kinematic approach that focuses on the forces acting on or inside the body. A kinematic approach that focuses on forces that cause movement is important to obtain knowledge that can be linked to actual training. This includes measuring the ground reaction force using a force plate and muscle contraction force using an electromyogram (EMG). Surface EMG data can noninvasively capture individual muscle activity during physical exercise [11]. Analysis and evaluation using EMG have been reported for the push movement of the hand rim, which is a basic element of wheelchair control. The push motion is performed by activating various muscles in a complex manner, and it is possible to evaluate the magnitude of the force exerted by observing the magnitude of the action potential of each muscle using surface myoelectricity. In addition, it is possible to evaluate the adjustment ability and smoothness of the push motion by looking at the target pattern [12,13,14,15].

However, EMGs are generally used under conditional control due to the limitation of body movements caused by attaching electrodes, devices, and cables to the body [16]. In addition, electrodes can shift position due to intense movements, which increases noise [17]. Thus, the development of a small wireless electromyogram [18] and an electrode [17] that does not come off easily using conductive gel has been reported. However, these EMGs are very expensive; thus, their accessibility is limited [19].

As a result, a method that can easily measure myoelectric potential at a low cost is expected. With the development of machine learning, markerless pose estimation using cameras has become possible [20,21]. This has eased the distance limitation from the camera to the subject, making it possible to estimate the posture of multiple people at relatively medium to long distances and to analyze movements during a game, where it is difficult to attach markers or sensors to the body. In the field of sports, markerless posture estimation using a camera has been applied to motion analysis and skill evaluation [22]. However, there are few previous studies that have used cameras for estimating muscle potential. The purpose of this study is to estimate muscle potential, which is kinematic information, using a video camera and an inertial sensor, both of which are increasingly used in the field of athletics as a kinematic approach. Once a training model is created, subsequent EMG potentials of the same person can be measured easily and inexpensively. Video cameras are unconstrained, and inertial sensors are small and lightweight; thus, such devices would not interfere with body movements. In addition, kinematics and kinetics information can be measured simultaneously; thus, this would be an extremely efficient measurement method. If we can establish such a data collection method, we can contribute to the development of new coaching and training concepts.

## 2. Related Work

In the sports field, many studies have implemented machine learning methods using inertial sensors and computer vision data as inputs [23]. For example, the use of support vector machines [24], logistic models [25], and hidden Markov models [26] has been reported. However, inertial sensor and computer vision data for sports movements are high-dimensional and contain noise; thus, learning is ineffective in many cases with such machine learning methods [27].

**Figure 1:**
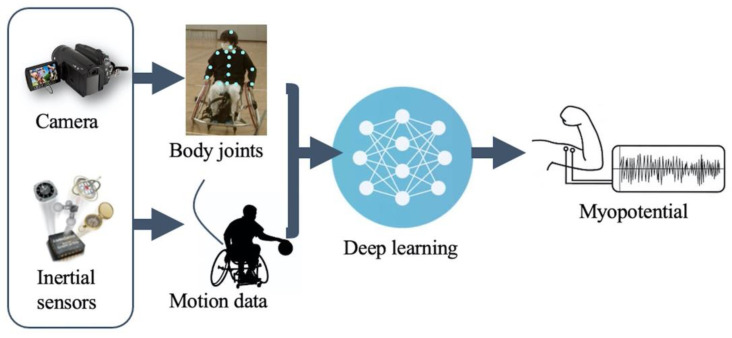
Outline of the proposed method.

Thus, deep learning methods have been reported in recent years [28,29]. Deep learning is based on a neural network, which is a system that imitates the mechanism of human nerve cells [30]. Using a multilayer neural network, features contained in the data can be learned step by step; thus, it is possible to automatically extract features from the data acquired by inertial sensors [31]. This allows us to avoid some data preprocessing and feature extraction processes that are required by nondeep machine learning techniques. However, to the best of our knowledge, few previous studies have reported myoelectric potential estimation methods for wheelchair operation using video cameras and inertial sensors. Therefore, we have developed a method to estimate myoelectric potential by applying a deep learning method to inertial sensor and computer vision data. Figure 1 shows an outline of the proposed method.

## 3. Proposed Method

**Figure 2:**
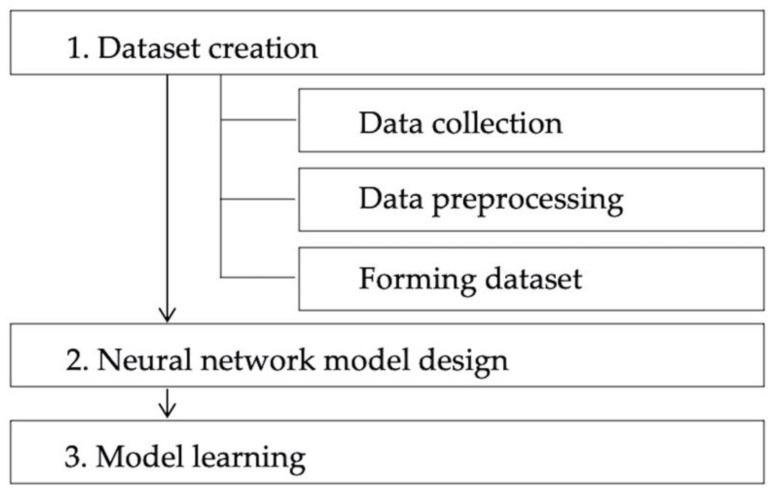
Steps to develop the myopotential estimation model.

In this section, we first describe our data collection, data preprocessing, and dataset formation processes. Then, the proposed myoelectric potential estimation model is described. Finally, we describe model learning and present an evaluation. Figure 2 shows the procedure used to develop a myoelectric potential estimation model.

### 3.1. Dataset Creation

#### 3.1.1. Data Collection

The data collection process involved synchronous measurement of myoelectric potential during wheelchair operation and capturing camera images and inertial sensor data. The subjects included seven healthy male subjects. (age: 22.9 ± 0.6 years; height: 171.7 ± 5.3 cm; weight: 64.2 ± 6.8 kg). Note that all subjects were right-handed. In addition, the subjects did not use a wheelchair on a daily basis but participated in the test after sufficient training. The measurement location was a gymnasium with a wooden floor.

**Figure 3:**
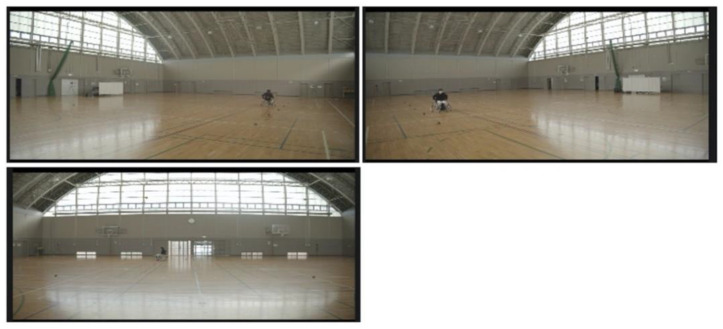
Example of measured camera images.

**Figure 4:**
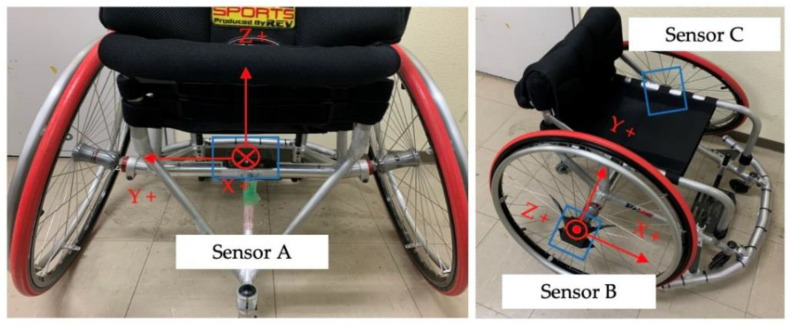
Example of measured camera images.

The measurement instruments included cameras (Pocket Cinema Camera 4K by Blackmagic Design Pty. Ltd., Port Melbourne, Australia), inertial sensors (IMS-SD, Tec Gihan Co., Ltd., Kyoto, Japan), and electromyograms (Polymate Pro MP6000, Miyuki Giken Co., Ltd., Tokyo, Japan) Met. Here, three cameras were used, and the cameras were installed such that the entire measurement field (length 10 m, width 10 m) could be seen. The sampling frequency was 60 Hz, and the image resolution was 4K. The three cameras input external triggers and measured them synchronously. Figure 3 shows images captured simultaneously by the three cameras. Figure 4 shows the installation position of the inertial sensor. The inertial sensor was equipped with a three-axis accelerometer, a three-axis angular velocity sensor, and a three-axis geomagnetic sensor. The coordinate system of the sensor was a right-handed system. Sensor A was installed at the center of the axle. The front direction of the wheelchair was the X-axis positive direction, and the vertical upward direction was the Z-axis positive direction. Sensor B was installed at the center of the right wheel, and sensor C was installed at the center of the left wheel. The vertical upward direction relative to the surface of the wheel was defined as the Z-axis positive direction. It was recorded at a sampling frequency of 1000 Hz. The inertial sensor has a resolution of 12 bits and a dynamic range of ±16 G, and the angular velocity sensor has a resolution of 16 bits and a dynamic range of ±2000 degree per second (dps).

**Figure 5:**
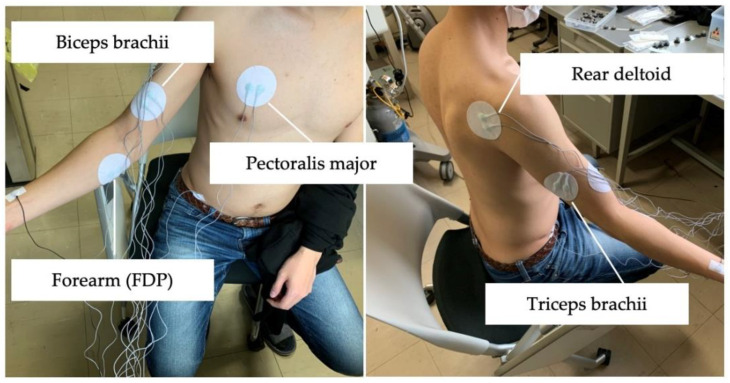
Attachment position for myopotential.

Figure 5 shows where the myoelectric potential is attached. The measurement points were the flexor digitorum profundus muscle, the biceps brachii muscle, the triceps brachii muscle, the posterior deltoid muscle, and the right pectoralis major muscle. Measurements were performed using active electrodes with a center distance of 2 cm between the electrodes. Measurements were recorded at a sampling frequency of 1000 Hz. Based on interviews with experts familiar with wheelchair sports, the target movements considered in this study included three-intensity straight running, zigzag running, 90-degree turns, 180-degree turns, and 360-degree turns, covering important chair work movements in wheelchair sports. Here, each subject performed a wheelchair movement for approximately five to eight minutes by mixing these movements. The wheelchair used in this study was a tennis wheelchair (BWZ, OX Engineering Co., Ltd., Chiba, Japan).

#### 3.1.2. Data Preprocessing

**Figure 6:**
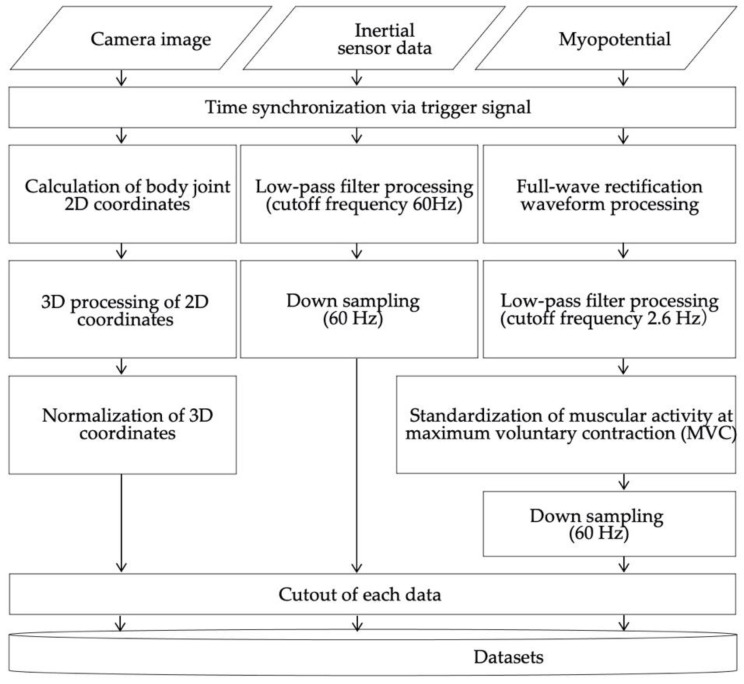
Procedure for preprocessing and formation of datasets.

**Figure 7:**
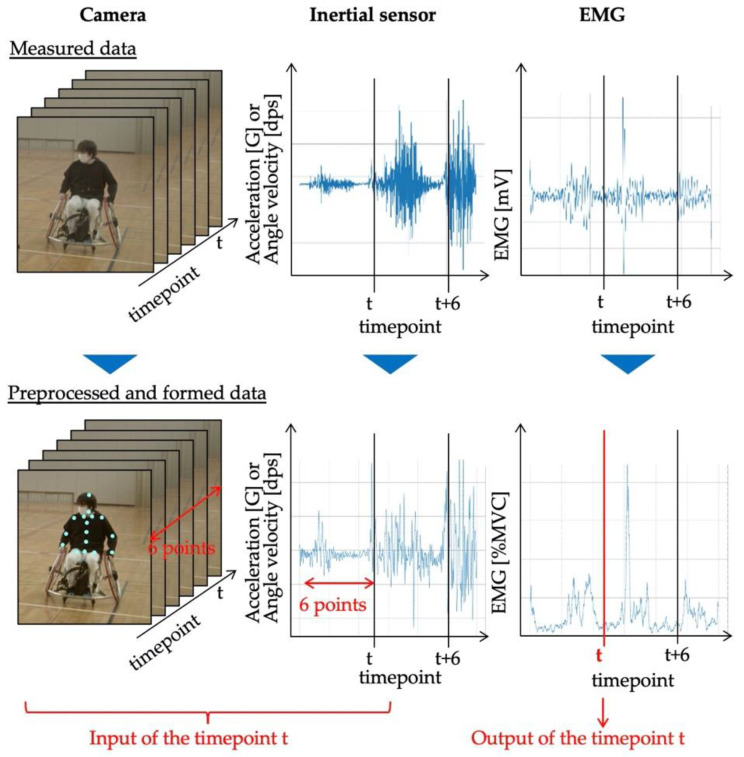
Sample data of the preprocessing and formation of datasets.

**Figure 8:**
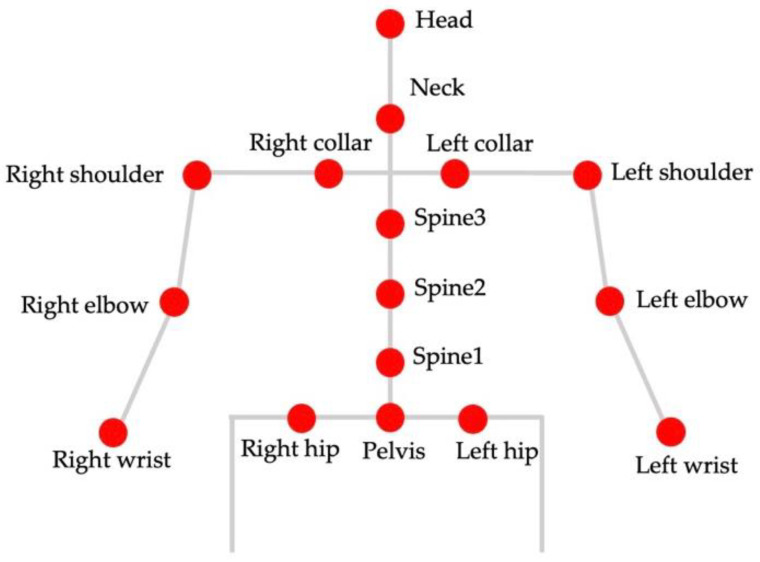
Body joints calculated from the camera image.

Here, we describe data preprocessing and dataset construction. Figure 6 shows the procedure used to create a dataset. Figure 7 shows example data used in data preprocessing and dataset construction. First, the data of each measurement instrument were time-synchronized by the external trigger signal input at the time of data collection. The images acquired by the cameras were processed as follows. First, the two-dimensional coordinates of the body joints of the person in the image were calculated from the camera image using Open Pose [32]. Of the three cameras, the camera image in which the subject was most prominent in the same time frame was used. In this study, we calculated the key point coordinates of the upper body, which are expected to affect the wheelchair rowing motion. Figure 8 shows the joint sites considered in this study. Next, the 3D coordinates were calculated from the calculated 2D coordinates of the body joints using a pose-baseline [33].

Finally, the calculated 3D coordinates of the body joints were normalized by converting them to a local coordinate system with the key point of the pelvis as the origin. Here, the midpoint between the right and left hips was the origin, and the vertical upward direction to the ground was the Z-axis positive direction. The plane that passes through the origin and is horizontal to the ground was defined as the XY plane. The line connecting the right and left hips was projected onto the XY plane as the Y-axis, and the direction from the right hip to the left hip was the positive direction. Note that this was a right-handed coordinate system. In addition, this process was repeated for each frame. The data acquired by the inertial sensor were processed as follows. The three-axis acceleration data of inertial sensor A were used to express the moving acceleration of the wheelchair, and the three-axis angular velocity data of inertial sensors B and C were used to express the rotation of the wheel. A high-pass filter with a cutoff frequency of 60 Hz was applied to the acceleration and angular velocity data to reduce high-frequency component noise. Then, to unify the sampling frequency of the data of each measurement instrument, we down-sampled it to 60 Hz.

The data recorded by the electromyogram were processed as follows. As a full-wave rectification smoothing process, the absolute value of the amplitude was obtained, and a low-pass filter with a cutoff frequency of 2.6 Hz was applied. To identify an appropriate cutoff frequency, we referred to the report by Yoshida and Terao [34]. Next, the percent maximum voluntary contraction (% MVC) was derived by normalizing the maximum myoelectric potential of the myoelectric potential data for each subject. Finally, to unify the sampling frequency of the data of each measurement instrument, we down-sampled it to 60 Hz.

#### 3.1.3. Dataset Construction

Here, we describe the dataset construction process. The input data that served as explanatory variables were 3D coordinate data for 16 body joints (48 dimensions in total), 3D acceleration data for wheelchairs, and the three-axis angular velocity data for the left and right wheels (six dimensions in total), for a total of 57 dimensions. The output value (i.e., the objective variable) was each myoelectric potential data point. Here, the data structure of time point t was the data of the input value obtained by cutting out the data of window width w points before time point t and the data of the output value obtained by cutting out the data of time point t. Next, time t was slid by slide width p, and the input and output data were cut out again. A dataset was constructed by repeating this process. In this study, the window width w and slide width were set to six. The constructed dataset included approximately 3800 data points for each subject.

### 3.2. Neural Network Model Design

A neural network model designed for myoelectric potential estimation is described in the following. Long Short-Term Memory (LSTM) [35] is generally used to extract features from time-series data. However, when attempting to recognize time-series data derived from muscle activity represented by an electrocardiogram, the one-dimensional convolutional neural network (1D-CNN) [36], which exhibits high performance in terms of feature extraction and learning short-time data, is better. Valid cases have been reported previously [37,38]. Therefore, a 1D-CNN was adopted in this study.

**Figure 9:**
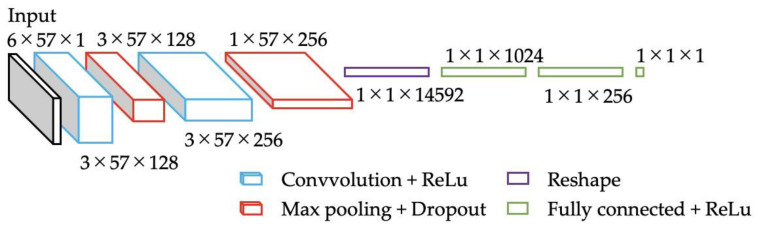
The architecture of the designed model (not to scale).

Figure 9 shows the architecture of the proposed model. The input to this model was a 6 × 57 size matrix. Next, it was connected to the convolutional layer for feature extraction. Here, the Relu function was adopted as the activation function. Next, we connected it to the max. pooling layer, which takes the maximum value in the kernel. Then, to avoid overfitting, we included a dropout layer [39] with a dropout rate of 0.5. This process was repeated twice. Note that the kernel sizes were set to 1 × 2 and 1 × 3, and the number of channels was changed to 128 and 256. As a result, the data of each dimension were convolved in the time direction, and feature quantity extraction was in progress. The data of each dimension extracted by the 1D-CNN were reshaped and then connected to the fully-connected layer. Here, the Relu function was employed as the activation function. Note that the fully-connected layer was repeated three times. Finally, the one-dimensional value was output, i.e., the myoelectric potential of a single target site.

### 3.3. Model Learning

The deep learning model described in Section 3.2 was implemented using Python and TensorFlow. Note that this was an in-subject model. The data for each subject were randomly divided into learning and evaluation data at a ratio of 8:2. In addition, 20% of the learning data were used as the validation data to avoid overfitting. The number of epochs was set to a maximum of 30,000, and the model with the parameters that had the smallest MSE (Mean Squared Error) in the validation data was adopted. Adam [40] was used as the optimal function, and the initial learning rate was set to 0.01. The batch size was set to 32, referring to a previous report [41]. The model was learned for each myoelectric potential site of each subject. For learning, we used an NVIDIA Tesla V100 on Google Colaboratory.

## 4. Results

Each trained model was applied to the evaluation data to evaluate the accuracy of myoelectric potential estimation. Here, the Spearman correlation coefficient between the measured EMG value and the value estimated by the proposed was obtained for each myoelectric site for each subject, and the significance of the correlation coefficient was tested. Table 1 shows the correlation coefficient between the measured and estimated values for each muscle site for each subject. As can be seen, all correlation coefficients were significant at the 0.1% level. The results of the estimation model for each subject and each myoelectric potential site exhibited a correlation coefficient of 0.5 or greater (*p* < 0.001). Thus, we confirmed that the proposed model can estimate a value with a positive correlation with the measured EMG value. By comparing the average values of the correlation coefficients for each subject, we found that the minimum and maximum values were 0.52 and 0.75, respectively. Although there were some differences depending on the subjects, all subjects had a correlation coefficient of 0.5 or greater. Similarly, by comparing the average values of the correlation coefficients for each myoelectric site, we found that the minimum and maximum values were 0.51 and 0.75, respectively. Here, although some differences were observed depending on the target myoelectric site, the correlation coefficient was 0.5 or greater in all cases.

The average and standard deviation of the absolute errors of the estimates obtained by the proposed model when the measured EMG values were taken as true values were calculated. Table 2 shows the average and standard deviation of the absolute error for each muscle site for each subject. As can be seen, the mean absolute error was 0.96 at the minimum and 7.78 at the maximum. Here, the range of data that can be taken by the true value was 0% to 100% MVC; thus, the maximum prediction error was 7.78%.

**Figure 10:**
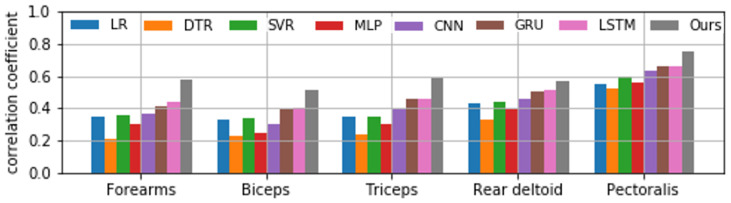
Comparison of correlation coefficients for each model.

Figure 10 shows the comparison of the correlation coefficients between the proposed method and other time-series data modeling algorithms. The models to be compared are conventional machine learning methods such as Linear Regression (LR) [42], Decision Tree Regression (DTR) [43], Support Vector Regression (SVR) [44], and Multilayer Perceptron (MLP) [45]. As a deep learning model, we used the widely used Convolutional Neural Network (CNN) [46]. In addition, we used the Gated Recurrent Unit (GRU) [47], which is widely used for extracting features from time series data, and the LSTM [35]. The proposed model showed the highest correlation among all models for all measurement sites. We showed the usefulness of the proposed model for the problem of estimating muscle potential from camera images and inertial sensors.

## 5. Discussion

As shown in Table 1, we found that the results of the proposed estimate model for each subject and each myoelectric potential site had a correlation coefficient of 0.5 or greater (*p* < 0.001), and the proposed model had a positive correlation with the measured EMG value. These results confirm that the value could be estimated. The evaluation data included data acquired during various chair work movements that occur in wheelchair sports; thus, we believe that the proposed model exhibits good generalizability for various chair work movements.

In addition, we confirmed that the average value of the correlation coefficient value for all subjects was 0.5 or greater. In addition, the proposed model was able to estimate a value having a positive correlation with the measured EMG value. Thus, the proposed method can be applied to various subjects. Similarly, the average correlation coefficient value for each myoelectric site achieved a correlation coefficient of 0.5 or greater for all myoelectric sites, and the proposed model could estimate a value that had a positive correlation with the measured EMG value. Thus, we have confirmed that the proposed method can handle various myoelectric sites.

In a previous study [48], the myoelectric potential during a dynamic loading task was estimated by solving the inverse dynamics and optimization problems using optical motion capture data and electromagnetic tracker data as inputs. It was reported that the deltoid muscle demonstrated an accuracy of 0.53 with the measured EMG, and the biceps brachii showed an accuracy of 0.61 with the measured EMG. The target motion in the current study is different from that of the previous study; thus, it is not possible to make a general comparison. However, the proposed model obtained the same degree of accuracy as the previous study using a simple measuring instrument.

The maximum prediction error of the proposed method was 7.78%. Similar to this study, in a previous study where kinematic data were estimated from kinematic data using deep learning, the results of estimating the skeletal muscle forces of the rectus femoris, soleus, and tibialis anterior muscles for walking movements indicated that the prediction error was less than 10% [49]. Note that we cannot make a general comparison with previous studies because the input kinematic data were acquired using a motion capture system, and the motions and myoelectric sites of the subjects differ; however, a simple measuring device was used in our study. Thus, we consider that the same degree of accuracy was obtained.

Since the myoelectric potential can be estimated without limiting the movement of the body by a simple method using only the camera image and the inertial sensor data, it is considered that it can be applied to the performance evaluation of wheelchair sports.

To estimate muscle potential, muscle activity, muscle strength, and joint torque accurately, the three-dimensional posture data of a person acquired using a motion capture system and mechanical data generated during exercise are required [49]. To acquire mechanical data while operating a wheelchair, a wheelchair [50] equipped with a torque meter that can measure the torque generated on the wheels via push operations is required. However, such wheelchairs are custom products and are not widely used; thus, so it is extremely difficult to acquire such wheelchairs. Therefore, in this study, we thought that the wheelchair would move as a result of the movement of wheelchair operation, and we used the wheelchair acceleration and angular velocity data obtained from the inertial sensor as pseudo mechanical data. As a result, the input values can be used as the three-dimensional posture data of the person during wheelchair operation and the mechanical data generated by movement. Thus, we believe that the proposed method contributes to myoelectric potential estimation. The proposed model is a unique approach that effectively exploits the specific characteristics of wheelchair sports.

In the field of wheelchair sports, running tests (e.g., straight run, turn, and zigzag run) on a defined course have been frequently conducted for the purpose of agility tests and wheelchair work skill evaluation, but the evaluation has been limited to running time only. Due to the limitation of high expertise and the high price of the measurement equipment, kinematic and muscle activity evaluations have not been conducted. The proposed model made it possible to estimate muscle potential using only simple devices, such as cameras and inertial sensors. Therefore, in the running test, it became possible to evaluate skills not only in terms of running time, which is the result of movements, but also the process of movements, especially the process of force generations. By evaluating the process of movements, it is possible to provide specific coaching. In addition, the proposed model makes it possible to assess the muscle fatigue caused by wheelchair operations. 

The limitations of the proposed method are described as follows. As mentioned previously, the proposed method was designed based on the premise that the acceleration and angular velocity data of the wheelchair obtained from the inertial sensor are used as pseudo mechanical data. Therefore, if the movement is unrelated to the wheelchair operation, e.g., the swing movement of a tennis racket or the shooting movement of a basketball, the acceleration and angular velocity data of the wheelchair obtained by the inertial sensor do not change; thus, the myoelectric potential will be incorrect. In addition, as basic research, we created a model of basic wheelchair movements with the advice of experts. 

However, it is difficult to apply the proposed model to actual competition scenes, because competition-specific movements are added to the basic wheelchair manipulation movements. For example, if a wheelchair is operated while holding a tennis or badminton racket, the EMGs may change. To solve this problem, we can collect additional EMGs during competition-specific motions and transfer learning the proposed model to cope with competition-specific motions. In order to solve this problem, we have been developing a model for estimating EMGs corresponding to competition-specific motions with the cooperation of Paralympic athletes playing wheelchair tennis and wheelchair badminton. In this study, we created a model to estimate the EMGs for each individual. Therefore, it is necessary to collect data using EMG for each person and use data as training to create the EMG estimation model. This point may be a barrier to the widespread use of this method in sports fields. The characteristics of EMGs, which are biometric information, differ slightly from person to person. Therefore, we will collect EMG data from a large number of subjects during wheelchair operations to expand the dataset. We will train the proposed model using the dataset and develop an EMG estimation model considering individual differences.

**Figure 11:**
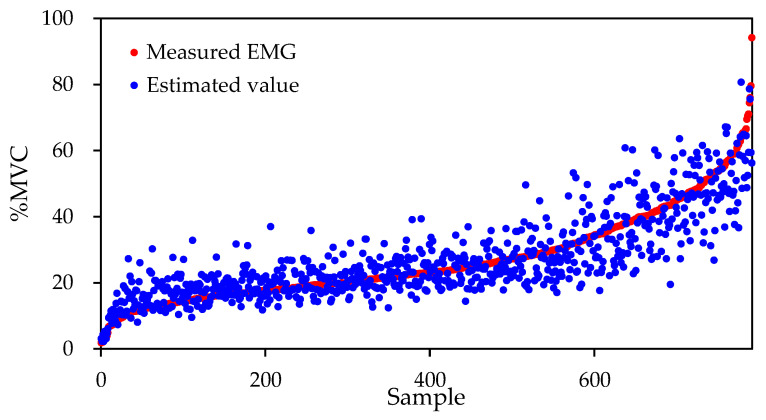
Plots of measured EMG and estimated value of the pectoralis in subject 1.

Figure 11 shows a plot of the electromyographic measurements and the estimated values obtained by the proposed model for the pectoralis major muscle of subject 1. Here, the horizontal axis plots the results of each sample such that the EMG measurement values are shown in ascending order. The vertical axis is the % MVC of the myoelectric potential. As shown in Figure 11, when the % MVC value is large, i.e., during a high-intensity push operation, the difference between the plots of the measured EMG values and the estimated values is large; thus, the estimation accuracy is poor. This tendency was also confirmed in other subjects and other myoelectric sites. It is considered that this is because the ratio of high-strength push motion was smaller than that of low-strength push motion in the data collection test conducted in this study. In other words, the feature quantity extraction effective for estimating the high-intensity push motion did not proceed in the learning process of the model because the data of the high-intensity push motion were scarce. To increase the proportion of high-intensity push motion data, data expansion by signal processing and additional data acquisition processes are required, which we plan to investigate in the future.

In the proposed model, a simple method was used to calculate a subject’s three-dimensional posture information from a camera image [32,33]. Compared to optical motion capture, the position accuracy of the three-dimensional posture is low. Thus, we expect to improve the accuracy of myoelectric potential estimation by improving the method used to calculate the 3D posture information from the camera images, which will improve position accuracy.

This study was the first attempt to estimate EMGs during wheelchair operation using the camera and inertial sensors. As a proof of concept, we showed that we applied deep learning to this estimation task and achieved a model with a moderate correlation to EMG measurements. In particular, we showed the usefulness of a deep learning model with 1D-CNN and fully connected layers. We believe that this realized effective feature extraction. However, the performance of a 1D-CNN may be inferior to that of LSTM [35] when long-term time information is required by estimating myoelectric potential because the time width that can be considered is fixed by the window size. Therefore, in the future, we will consider designing a neural network model that fuses the two methods, e.g., by applying the 1D-CNN for data close to the input and applying LSTM in the higher-order region.

Recall that the proposed myoelectric potential estimation model is an in-subject model. In other words, the model ignores individual differences. By training the proposed model on a dataset with an increased number of subjects and additional training data, we believe that an intersubject model that considers individual differences can be realized in the future.

## 6. Conclusions

In order to realize a simple method for measuring EMGs as a proof of concept study, we proposed a model for estimating EMGs during wheelchair operations using deep learning from camera images and inertial sensor data. We designed a deep learning model using a 1D-CNN and trained the model using a collected dataset. We found that the proposed model can estimate myoelectric potential that correlates with the measured EMG values (showing a correlation coefficient of 0.5 or greater). In addition, we achieved the same level of accuracy as a previous myoelectric potential estimate method that acquires values by a motion capture system using inverse dynamics and optimization problems. We have demonstrated a new approach to estimating myoelectric potential during wheelchair operation using only kinematic data and deep learning. We have also suggested the possibility of estimating EMG using only camera image and inertial sensor data without using the EMG. Since this method can estimate the myoelectric potential without limiting the movement of the body, it is considered that it can be applied to the performance evaluation of wheelchair sports.

In the future, we plan to improve the accuracy of myoelectric potential estimation and to develop an estimation model that considers individual differences.

## Figures and Tables

**Table 1:** The correlation coefficient between measured EMG values and proposed model estimated values. All correlation coefficients in the table are significant at a 0.1% level.

	Forearms	Biceps	Triceps	Rear Deltoid	Pectoralis	Micro Average
Subject 1	0.74	0.64	0.57	0.84	0.86	0.73
Subject 2	0.68	0.73	0.60	0.88	0.88	0.75
Subject 3	0.41	0.40	0.49	0.41	0.92	0.53
Subject 4	0.54	0.50	0.63	0.32	0.87	0.57
Subject 5	0.60	0.33	0.64	0.37	0.64	0.52
Subject 6	0.60	0.39	0.64	0.57	0.47	0.53
Subject 7	0.49	0.55	0.60	0.56	0.58	0.56
Micro Average	0.58	0.51	0.59	0.57	0.75	-

**Table 2:** Mean and standard deviation of the absolute error between measured EMG values and proposed model estimated values. The unit is %MVC.

	Forearms	Biceps	Triceps	Rear Deltoid	Pectoralis
Subject 1	5.11 ± 7.06	7.21 ± 6.56	3.83 ± 5.75	5.53 ± 5.12	5.15 ± 5.15
Subject 2	5.69 ± 6.70	6.24 ± 6.18	6.89 ± 6.12	4.29 ± 5.15	4.97 ± 6.05
Subject 3	5.75 ± 6.12	7.47 ± 6.88	7.10 ± 9.21	5.40 ± 7.66	4.57 ± 4.27
Subject 4	6.37 ± 6.51	6.98 ± 6.06	5.63 ± 7.28	2.47 ± 4.67	4.36 ± 4.11
Subject 5	4.58 ± 5.46	0.96 ± 4.20	4.76 ± 5.65	4.38 ± 7.80	5.69 ± 7.53
Subject 6	6.72 ± 7.69	5.72 ± 7.04	5.55 ± 8.68	7.78 ± 8.34	5.65 ± 8.80
Subject 7	4.73 ± 6.88	5.83 ± 7.05	5.05 ± 8.14	6.62 ± 6.38	4.65 ± 5.45

## Data Availability

All available data can be obtained by contacting the corresponding author.
